# The H_2_S-Donor Erucin Exhibits Protective Effects against Vascular Inflammation in Human Endothelial and Smooth Muscle Cells

**DOI:** 10.3390/antiox10060961

**Published:** 2021-06-15

**Authors:** Alma Martelli, Eugenia Piragine, Era Gorica, Valentina Citi, Lara Testai, Eleonora Pagnotta, Luca Lazzeri, Nicola Pecchioni, Valerio Ciccone, Rosangela Montanaro, Lorenzo Di Cesare Mannelli, Carla Ghelardini, Vincenzo Brancaleone, Lucia Morbidelli, Vincenzo Calderone

**Affiliations:** 1 Department of Pharmacy, University of Pisa, Via Bonanno 6, 56126 Pisa, Italy; eugenia.piragine@farm.unipi.it (E.P.); era.gorica@phd.unipi.it (E.G.); valentina.citi@unipi.it (V.C.); lara.testai@unipi.it (L.T.); 2 Interdepartmental Research Center “Nutrafood: Nutraceutica e Alimentazione per la Salute”, University of Pisa, 56126 Pisa, Italy; 3 Interdepartmental Research Center “Biology and Pathology of Ageing”, University of Pisa, 56126 Pisa, Italy; 4 Research Centre for Cereal and Industrial Crops, CREA Council for Agricultural Research and Economics, Via di Corticella 128, 40134 Bologna, Italy; eleonora.pagnotta@crea.gov.it (E.P.); luca.lazzeri@crea.gov.it (L.L.); 5 Research Centre for Cereal and Industrial Crops, CREA Council for Agricultural Research and Economics, S.S. 673 Km 25,200, 71122 Foggia, Italy; nicola.pecchioni@crea.gov.it; 6 Department of Life Sciences, University of Siena, Via A. Moro 2, 53100 Siena, Italy; ciccone3@student.unisi.it (V.C.); morbidelli@unisi.it (L.M.); 7 Department of Science, University of Basilicata, Via dell’Ateneo lucano, 10, 85100 Potenza, Italy; rosangela.montanaro@unibas.it (R.M.); vincenzo.brancaleone@unibas.it (V.B.); 8 Department of Neuroscience, Psychology, Drug Research and Child Health–NEUROFARBA–Section of Pharmacology and Toxicology, University of Florence, 50139 Florence, Italy; lorenzo.mannelli@unifi.it (L.D.C.M.); carla.ghelardini@unifi.it (C.G.)

**Keywords:** vascular inflammation, endothelial permeability, H_2_S-donor, erucin, isothiocyanate, *Eruca sativa* Mill., vascular wall, hydrogen sulfide

## Abstract

Preservation of vascular wall integrity against degenerative processes associated with ageing, fat-rich diet and metabolic diseases is a timely therapeutical challenge. The loss of endothelial function and integrity leads to cardiovascular diseases and multiorgan inflammation. The protective effects of the H_2_S-donor erucin, an isothiocyanate purified by *Eruca sativa* Mill. seeds, were evaluated on human endothelial and vascular smooth muscle cells. In particular, erucin actions were evaluated on cell viability, ROS, caspase 3/7, inflammatory markers levels and the endothelial hyperpermeability in an inflammatory model associated with high glucose concentrations (25 mM, HG). Erucin significantly prevented the HG-induced decrease in cell viability as well as the increase in ROS, caspase 3/7 activation, and TNF-α and IL-6 levels. Similarly, erucin suppressed COX-2 and NF-κB upregulation associated with HG exposure. Erucin also caused a significant inhibition of p22phox subunit expression in endothelial cells. In addition, erucin significantly prevented the HG-induced increase in endothelial permeability as also confirmed by the quantification of the specific markers VE-Cadherin and ZO-1. In conclusion, our results assess anti-inflammatory and antioxidant effects by erucin in vascular cells undergoing HG-induced inflammation and this protection parallels the preservation of endothelial barrier properties.

## 1. Introduction

Protection of the vascular wall from progressive degenerative processes represents one of the most important challenges in cardiovascular pharmacology. Indeed, the integrity of the vascular wall is often undermined by both persistent oxidative stress and a low-grade, chronic and subclinic inflammation. This pathological condition, which is defined as “vascular inflammation”, can be induced by several factors, such as ageing, fat-rich diet and/or development of metabolic diseases (i.e., metabolic syndrome, diabetes or an alteration in serum lipid concentrations) [1]. First, the loss of endothelium and smooth muscle function leads to cardiovascular consequences, including the impairment of vascular tone, the increase in platelet aggregation and macrophage infiltration in the onset of atherosclerotic plaques. Moreover, the loss of vascular wall integrity also leads to the condition of “leaky” vascular tree, which could spread inflammation in several districts [2]. Combined endothelial and smooth muscle dysfunction affects many people (especially those over 40) in Western society and, if not adequately prevented, it could lead to clinically relevant cardiovascular diseases such as hypertension, cognitive impairment, myocardial infarct, stroke and erectile dysfunction [3]. In this scenario, the use of nutraceutical compounds may represent a valuable approach, which seems to be particularly appropriate for a preventive strategy. Indeed, a large variety of botanicals present in food, or better dosed and administered as supplements, are endowed with interesting anti-inflammatory and antioxidant activities that could be used for the prevention of endothelial dysfunction derived from vascular inflammation. For example, dietary molecules that slowly release hydrogen sulfide (H_2_S), such as garlic polysulfides [4] or isothiocyanates from brassica vegetables [5], seem to be the most promising compounds with demonstrated pre-clinical mechanisms of action and epidemiological evidence [6]. Indeed, the gasotransmitter H_2_S, released by slow H_2_S-donors (such as isothiocyanates [7,8]), exhibits well-known antioxidant properties, which are mainly related to the activation of antioxidant pathways, such as the Nrf2/ARE (nuclear erythroid 2-related factor 2/antioxidant response element) axis [9]. Moreover, H_2_S promotes significant anti-inflammatory effects, mainly through inhibition of the pro-inflammatory transcription factor NF-ĸB (Nuclear factor kappaB) and prevention of mast cell degranulation [10,11]. Furthermore, many pathological conditions (e.g., atherosclerosis, diabetes) deeply correlate with vascular inflammation and can be linked to H_2_S deficiency, since its endogenous biosynthesis dramatically drop down in such disorders [12,13,14]. Therefore, restoring the physiological levels of the H_2_S with exogenous donors (i.e., isothiocyanates) might contribute to prevention and/or treatment of several conditions associated with vascular inflammation.

Among the natural isothiocyanates, erucin is a promising candidate for nutraceutical purposes, as it is widely present in edible plants belonging to the *Brassicaceae* botanical family. Indeed, this aliphatic isothiocyanate is derived from both *Eruca sativa* Mill., e.g., rocket salad, and inter-conversion of sulforaphane (an isothiocyanate from *Brassica oleracea* L.), which occurs in vivo [15]. The appreciable oral bioavailability of erucin (as well as other natural isothiocyanates) after *Brassicaceae* consumption has been demonstrated in rodents as well as in humans [16,17]. Therefore, dietary consumption of erucin is reasonably devoid of possible toxic effects. Moreover, as reported in a previous work, erucin displayed features of a slow and smart (i.e., long-lasting and thiol-dependent) H_2_S-donor. In particular, the amperometric recording of H_2_S release from this aliphatic isothiocyanate led to the observation that, in the presence of l-Cysteine, the incubation of erucin was followed by a stable and long-lasting generation of H_2_S [18]. A slow and long-lasting H_2_S release from exogenous H_2_S-donors is likely to be more reliable than other compounds (i.e., sulfide inorganic salt) in mimicking the profile of endogenous H_2_S generation [12]. The chemical mechanism accounting for the generation of H_2_S from isothiocyanates has been recently described and is l-Cysteine-mediated [19].

More recently, erucin exhibited vasorelaxant and anti-hypertensive effects strictly related to the release of H_2_S in a gradual and physiological-like manner [20]. This study was carried out to evaluate the potential H_2_S-mediated protective effect generated by erucin in endothelial and vascular smooth muscle cells, as the key cell types modulating vessel wall homeostasis, exposed to inflammatory stimuli, aiming to identify a possible candidate to prevent vascular inflammation.

## 2. Materials and Methods

### 2.1. Chemicals and Reagents

Erucin was produced by myrosinase-catalyzed hydrolysis of glucoerucin isolated from *Eruca sativa* Mill. defatted seed meals according to Citi et al. [21]. Erucin was dissolved in dimethyl sulfoxide (DMSO; Merck KGaA, Darmstadt, Germany) and this solution (10^−2^ M) was freshly diluted in the appropriate culture medium.

An aqueous solution of d-(+)-Glucose (45% *w*/*w*; Merck KGaA, Darmstadt, Germany) was diluted before each experiment in culture medium up to a final concentration of 25 mM. CelLytic™ MT Cell Lysis Reagent, Fluoromount Aqueous Mounting Medium, and 3 kDa FITC-Dextran were obtained from Life Technologies (Carlsbad, CA, USA). Anti-VE-Cadherin was obtained from Cell Signalling (Danvers, MA, USA) and anti-ZO-1 was obtained from Life Technologies (Carlsbad, CA, USA). Anti-SOD-1, anti-iNOS, anti-NF-KB and anti-p22phox antibodies were obtained from Santa Cruz Biotechnology (Dallas, TX, USA). Anti-catalase and anti-β-actin were obtained from Merck KGaA, (Darmstadt, Germany).

### 2.2. Cell Cultures

Human aortic smooth muscle cells (HASMCs, Thermo Fisher Scientific, Waltham, MA, USA) were cultured in a basal medium (Medium 231, Thermo Fisher Scientific, Waltham, MA, USA) containing both Smooth Muscle Growth Supplement (SMGS, Thermo Fisher Scientific, Waltham, MA, USA) and a solution of antibiotics (100 units/mL penicillin plus 100 mg/mL streptomycin, Merck KGaA, Darmstadt, Germany). Human umbilical vein endothelial cells (HUVECs, Thermo Fisher Scientific, Waltham, MA, USA) were cultured in a basal medium (Medium 131, Thermo Fisher Scientific, Waltham, MA, USA) supplemented with fetal bovine serum 10% (FBS, Hyclone, Celbio, Milan, Italy), antibiotics (100 units/mL penicillin plus 100 mg/mL streptomycin), 1% L-glutamine, 10 U/mL heparin, epidermal growth factor (EGF, 10 ng/mL) and basic fibroblast growth factor (bFGF, 5 ng/mL). All supplements were purchased from Merck KGaA (Darmstadt, Germany). Cells were cultured in T-75 flasks at 37 °C in a CO_2_ (5%) incubator up to about 90% confluence before starting the experiments. For the experiments described in Section 2.7, Section 2.8 and Section 2.9, HUVECs (Promocell, Heidelberg, Germany) were cultured in endothelial growth medium (EGM-2), containing VEGF, R3-IGF-1, hEGF, hFGF, hydrocortisone, ascorbic acid, heparin and GA-1000 (Lonza, Basel, Switzerland), 10% FBS and 2 mM glutamine, 100 units/mL penicillin and 0.1 mg/mL streptomycin (Merck KGaA, Darmstadt, Germany). Cells were incubated at 37 °C in 5% CO_2_. HASMCs between passages 3 to 15 and HUVECs between passages 3 to 5 were used in the experiments. Control of mycoplasma was performed using frozen vials.

### 2.3. Measurement of Intracellular H_2_S Release in Endothelial Cells

HUVECs were seeded at 30,000 cells/well in a black 96-well plate coated with an aqueous solution of gelatin (1%) (Merck KGaA, Darmstadt, Germany). After 24 h, the medium was replaced with a freshly prepared solution of WSP-1 (Washington State Probe-1, 3′-methoxy-3-oxo-3Hspiro[isobenzofuran-1,9′-xanthen]-6′-yl-2(pyridin-2-yldisulfanyl benzoate); Cayman Chemical, Ann Arbor, MI, USA), 100 µM. WSP-1 is a highly sensitive, fluorescent dye which selectively reacts with intracellular H_2_S, releasing a fluorophore [22]. After an incubation period of 30 min (to allow cells to incorporate the probe), the supernatant was replaced with vehicle (1% DMSO) or erucin (30, 100 and 300 μM) diluted in standard buffer, pH 7.4 (composition: HEPES 20 mM, KCl 2 mM, NaCl 120 mM, MgCl_2_·6H_2_O 1 mM, glucose 5 mM and CaCl_2_·2H_2_O 2 mM). The well-described slow H_2_S-donor diallyldisulfide (DADS, Merck KGaA, Darmstadt, Germany) [23] was selected as the reference compound (300 µM). Change in fluorescence was monitored for 40 min every 5 min at λex = 465 nm, λem = 515 nm, using a multiwell plate reader (EnSpire; PerkinElmer, Waltham, MA, USA) [24].

### 2.4. Cell Viability

HASMCs were seeded at 10,000 cells/well in a 96-well plate. After 24 h, the culture medium was removed and cells were treated with erucin (0.3, 1 and 3 µM) or its vehicle (0.03% DMSO) for 1 h. Then, the growth medium was replaced with a serum-free culture medium supplemented with a solution of d-(+)-glucose at 25 mM concentration (high glucose, HG). Erucin (0.3, 1 and 3 µM) or its vehicle (0.03% DMSO) were added again for 72 h. HUVECs were seeded at 20,000 cells/well in a 96-well plate. The day after, the culture medium was removed and cells were treated with a solution of erucin (0.3, 1 and 3 µM) or its vehicle (0.03% DMSO) for 1 h. Then, a freshly prepared solution of d-(+)-glucose was incubated in each well for 24 h, without removing the culture medium. The final concentration of d-(+)-glucose in the well was 25 mM. At the end of the treatment, an aqueous solution of the soluble tetrazolium salt WST-1 (Roche, Basilea, Switzerland) was incubated (1:10) for 1 h at 37 °C in a CO_2_ incubator. Cell viability was spectrophotometrically assessed using the microplate reader EnSpire (PerkinElmer, Waltham, MA, USA) at λ = 495 nm.

### 2.5. Evaluation of Caspase-3/7 Activity

HASMCs were seeded at 10,000 cells/well in a 96-well black plate. After 24 h, the culture medium was removed and cells were treated with erucin (0.3, 1 and 3 µM) or its vehicle (0.03% DMSO) for 1 h. Then, the growth medium was replaced with a serum-free culture medium enriched with 25 mM d-(+)-glucose. Erucin (0.3, 1 and 3 µM) or its vehicle (0.03% DMSO) was added again for 72h. HUVECs were seeded onto a 96-well black plate (20,000 cells/well). After 24 h, the culture medium was removed and cells were treated with erucin (0.3, 1 and 3 µM) or its vehicle (0.03% DMSO) for 1 h. Then, a freshly prepared solution of 25 mM d-(+)-glucose was added to each well for 24 h, without removing the medium.

At the end of the treatment, the freshly mixed ApoONE^®^ Homogeneous Caspase-3/7 Buffer and Substrate (Promega, Madison, WI, USA) was incubated in the dark for 50 min at room temperature under continuous mixing, according to the manufacturer’s protocol. Caspase-3/7 activity was determined spectrofluorimetrically using the microplate reader EnSpire (PerkinElmer, Waltham, MA, USA) at λex = 488 nm and λem = 533 nm.

### 2.6. Measurement of Intracellular Reactive Oxygen Species (ROS) Production

HASMCs were seeded at 30,000 cells/well in a 96-well black plate coated with an aqueous solution (1%) of gelatin. After 24 h, the culture medium was removed and cells were treated with erucin (0.3, 1 and 3 µM) or its vehicle (0.03% DMSO) for 1 h. Then, the growth medium was replaced with a serum-free culture medium enriched with 25 mM d-(+)-glucose. Erucin (0.3, 1 and 3 µM) or its vehicle (0.03% DMSO) was added again for 72 h. HUVECs were seeded in a 96-well black plate (30,000 cells/well) coated with an aqueous solution (1%) of gelatin. After 24 h, the culture medium was removed and cells were treated with erucin (0.3, 1 and 3 µM) or its vehicle (0.03% DMSO) for 1 h. Then, a freshly prepared solution of d-(+)-glucose was added to each well, without removing the medium. The solution of d-(+)-glucose (25 mM) was incubated for 24 h. At the end of the treatment, an aqueous solution of the fluorescent probe 2′,7′-dichlorofluorescin diacetate (DCFDA, 20 µM; Merck KGaA, Darmstadt, Germany) was incubated at 37 °C for 45 min in a CO_2_ (5%) incubator in the dark. DCFDA is a non-fluorescent probe which is de-esterified inside cells, oxidized and converted into the highly fluorescent 2′,7′-dichlorofluorescein. Fluorescence values, corresponding to intracellular ROS production, were measured using the microplate reader EnSpire (PerkinElmer, Waltham, MA, USA) at λex = 500 nm and λem = 530 nm.

### 2.7. Endothelial Permeability

HUVECs (8 × 10^4^/insert) were seeded on gelatin-coated insert membranes (Corning, NY, USA) with 0.4 µm-diameter pores and grown in 12 multiwell plates for 72 h. Confluent monolayers were pre-treated with erucin (3 µM, 1 h), and then a solution of d-(+)-glucose or mannitol (25 mM, 24 h) was added where indicated. Over the treatments, the cells were maintained in medium with 10% FBS. FITC-Dextran (3 kDa, 10 μM) was used as a fluorescent probe of permeability. Every 15 min, fluorescence (485 and 535 nm excitation and emission, respectively) was measured by using a microplate reader (Tecan, Infinite 200 Pro, SpectraFluor, Männedorf, Switzerland) [25]. Data are reported as fluorescence intensity.

### 2.8. Western Blot

Subconfluent endothelial cells were seeded in 60 mm Petri dishes. After 24 h, cells were pre-treated with erucin (3 µM, 1 h) and then 25 mM d-(+)-glucose was added in medium containing 10% FBS for 24 h. At the end of stimulation, proteins were isolated and Western blots were performed as previously described [26]. Immunoblots were analyzed by densitometry using Image J 1.48v software (U.S. National Institutes of Health, Bethesda, MD, USA), and the results, expressed as arbitrary density units (A.D.U.) ± SD, were normalized toward β-actin.

### 2.9. Immunofluorescence Analysis

The cell–cell contact proteins vascular endothelial-cadherin (VE-Cadherin) and zonula occludens-1 (ZO-1) were visualized by immunofluorescence analysis. Then, 5 × 10^4^ HUVECs were seeded on 1 cm circular glass coverslips. After 24 h, confluent cells were washed and pre-treated with erucin (3 µM, 1 h) and then with 25 mM d-(+)-glucose for 24 h. Immunofluorescence analysis was performed as previously reported and images were taken using a confocal microscope [25]. Fluorescence intensity was measured using Fiji software.

### 2.10. Cytokine Array Panel

HASMCs and HUVECs were seeded at a density of 500,000 cells/well in a 6-well plate pre-coated with an aqueous solution (1%) of gelatin. After 24 h, cells were treated with erucin 3 µM or its vehicle (0.03% DMSO) for 1h before exposing cells to d-(+)-glucose 25 mM (as reported in Section 2.4). Cytokine determination was performed by using a commercially available kit (Abcam, Cambridge, UK) detecting 23 different cytokines. An array panel was developed according to manufacturer indications. HASMCs and HUVECs were homogenized following the treatments and were diluted to a final concentration of 250 µg/mL. A separate membrane was used for each treatment (vehicle, HG, HG + erucin 3 µM). Membranes, following overnight incubation with samples at 4 °C, were washed and incubated with a cocktail of biotin-conjugated anti-cytokine antibodies for 2 h at room temperature (RT) and were then washed again and incubated with HRP-conjugated streptavidin for 2 h at RT. Membranes were washed again and then chemiluminescence substrate was added. Dot-spot signals were captured by using the ChemiDoc Imaging System (BioRad, Hercules, CA, USA).

### 2.11. ELISA Assay

ELISA assays for interleukin 6 (IL-6) and tumor necrosis factor-alpha (TNF-α, ThermoFisher, Milan, Italy) were carried out in cell supernatants from HASMCs and HUVECs following treatments (vehicle, HG and HG + Erucin 3 µM) according to the manufacturer instructions. Briefly, 100 μL of samples, diluted standards, quality controls, and dilution buffer (blank) were added to a pre-coated plate with monoclonal anti-IL-6 or anti-TNF-α. After washing, 100 μL of biotin-labelled antibody was added for 1 h. The plate was washed and 100 μL of streptavidin–HRP conjugate was added and the plate was then incubated for a further 30 min period in the dark. The addition of 100 μL of the substrate and stop solution represented the last steps before the reading of absorbance, measured at 450 nm on a microplate reader (ThermoFisher, Milan, Italy).

### 2.12. Data Analysis

The results are reported as the mean ± SEM. One-way ANOVA followed by Bonferroni’s post hoc test was selected for statistical analysis. When appropriate, the Student’s *t* test has been also used. Statistical analysis was performed using GraphPad Prism 5.0 (La Jolla, CA, USA). Values were considered statistically different for *p* < 0.05. Results are derived from 6 independent experiments, each performed in triplicate.

## 3. Results

### 3.1. Intracellular H_2_S Release

**Figure 1:**
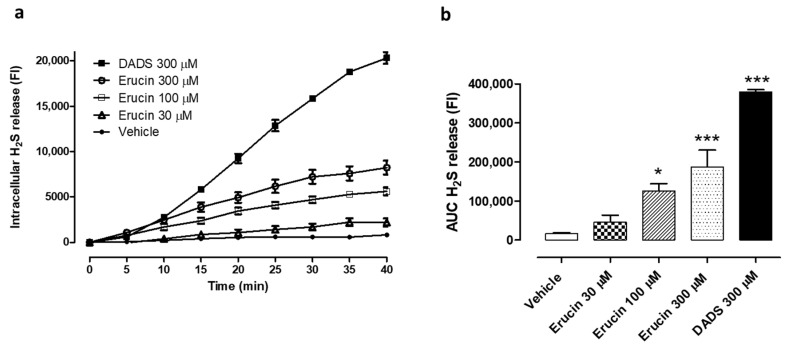
Evaluation of the H_2_S-releasing properties of erucin in relation to HUVECs. (**a**) Time course of the fluorometric recording of H_2_S released by vehicle (1% DMSO), erucin (30, 100 and 300 µM) or the reference H_2_S-donor diallyl disulfide (DADS, 300 µM). The increase in intracellular levels of H_2_S is expressed as fluorescence index (FI). (**b**) Histograms show the amount of H_2_S released by erucin (30, 100 and 300 µM), DADS 300 μM and vehicle (1% DMSO) expressed as area under curve (AUC). All the experiments were carried out in triplicate for a minimum of three times (*n* = 9). Data are shown as mean ± SEM. Statistical significance has been calculated by one-way ANOVA followed by Bonferroni post-test. * indicates a value statistically different from vehicle (* *p* < 0.05; *** *p* < 0.001).

As previously observed in HASMCs [20], erucin was also able to release H_2_S in HUVECs. In particular, the administration of three different concentrations of erucin (30, 100 and 300 µM) to HUVECs induced a slow and concentration-dependent increase in the intracellular fluorescence associated with the interaction between WSP-1 dye and H_2_S released by erucin (Figure 1a). This increase reached a plateau after about 40 min and, following the AUC quantification, the amount of H_2_S released by 300 µM erucin reached about 50% of the value recorded for cells incubated with the same concentration (300 µM) of the DADS, used as positive control (Figure 1b).

### 3.2. Evaluation of the Cell Viability Preservation against HG-Induced Cell Damage in HASMCs and HUVECs

**Figure 2:**
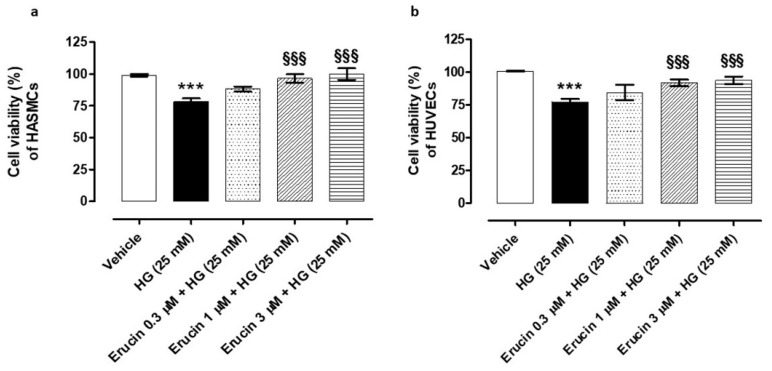
Preventive effects of erucin against high glucose-induced vascular cell death. Graphs show cell viability (%) of HASMCs (**a**) and HUVECs (**b**) exposed to high levels of d-(+)-glucose (HG, 25 mM). Pre-incubation with erucin (0.3, 1 and 3 µM) for 1h prevented HG-induced cell death in a concentration-dependent manner. All the experiments were carried out in triplicate for a minimum of three times (*n* = 9). Data are shown as mean ± SEM. Statistical significance has been calculated by one-way ANOVA followed by Bonferroni post-test. * indicates significant difference vs. vehicle (*** *p* < 0.001), while § indicates significant difference vs. HG (25 mM) (§§§ *p* < 0.001).

The protective effect of erucin against an inflammatory stimulus obtained by the administration of HG 25 mM was tested on both HUVECs and HASMCs. The HG-exposed HASMCs displayed a decrease in cell viability by 22% (% cell viability vs. control: 78.14 ± 2.67). This decrease was prevented by the pre-incubation of different concentrations of erucin (% cell viability vs. vehicle for 0.3 µM: 88.18 ± 1.99, for 1 µM: 96.56 ± 3.27 and for 3 µM: 99.95 ± 4.50) in a concentration-dependent manner (Figure 2a). At the same time, HG treatment in HUVECs induced a decrease in cell viability by 23% (% cell viability vs. control: 77.28 ± 2.19). Similarly, the pre-incubation with the different concentrations of erucin in HUVECs induced a concentration-dependent protective effect (% cell viability vs. vehicle for 0.3 µM: 84.29 ± 5.91, for 1 µM: 91.67 ± 2.26 and for 3 µM: 93.75 ± 2.91) (Figure 2b).

### 3.3. Anti-Apoptotic Effects of Erucin in Vascular Cells Exposed to HG

**Figure 3:**
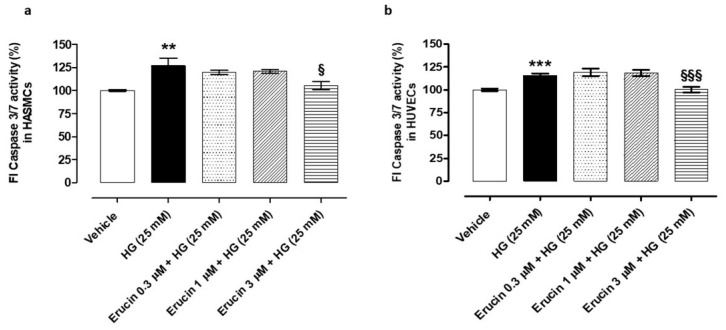
Anti-apoptotic effects of erucin in vascular cells exposed to high glucose. Histograms show the activity of the pro-apoptotic caspase 3/7 in both HASMCs (**a**) and HUVECs (**b**) treated with vehicle (0.03% DMSO) or increasing concentrations of erucin (0.3, 1 and 3 µM) for 1 h, which were then exposed to high glucose levels (HG). The highest concentration of erucin tested significantly prevented HG-induced caspase 3/7 activation in both vascular cell lines. All the experiments were carried out in triplicate for a minimum of three times (*n* = 9). Data are expressed as mean ± SEM. Statistical significance has been calculated by one-way ANOVA followed by Bonferroni post-test. * indicates statistical difference vs. vehicle (** *p* < 0.01; *** *p* < 0.001), while § indicates statistical difference vs. high glucose-treated cells pre-incubated with vehicle (§ *p* < 0.05; §§§ *p* < 0.001).

The administration of HG-induced apoptosis both in HASMCs and HUVECs. In HASMCs, HG evoked an increase in the fluorescence index (FI) linked to the caspase 3/7 activity by 26.88 ± 7.94% compared to vehicle-treated cells. Pre-incubation of erucin prevented this change, particularly at the top concentration used (% increase vs. vehicle-treated cells in: erucin 0.3 µM + HG-treated cells: 19.41 ± 2.33; erucin 1 µM + HG-treated cells: 20.64 ± 1.83; erucin 3 µM + HG-treated cells: 5.24 ± 4.22) (Figure 3a). In HUVECs, exposure to HG induced a significant increase in caspase 3/7 activation by 15.96 ± 1.95% vs. the vehicle-treated cells, while this effect was fully inhibited by the pre-incubation with erucin 3 µM (Figure 3b).

### 3.4. Preventive Effects of Erucin against HG-Induced Intracellular ROS Production

**Figure 4:**
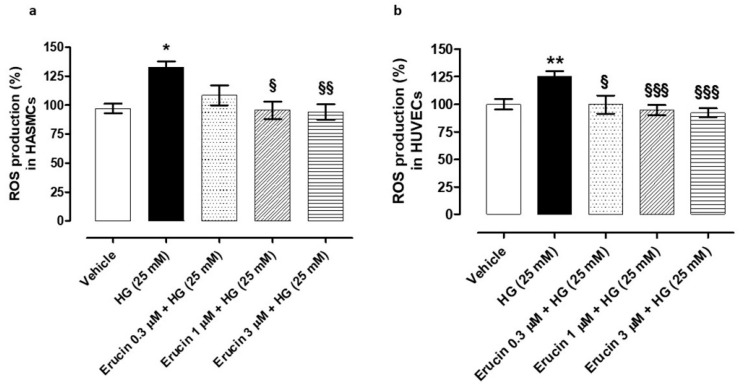
Evaluation of the preventive effects of erucin against glucose-induced intracellular ROS production. Histograms show the levels of intracellular ROS (%) in HASMCs (**a**) and HUVECs (**b**) treated with vehicle (0.03% DMSO) or increasing concentrations of erucin (0.3, 1 and 3 µM) for 1 h, which were then exposed to high glucose levels (HG, 25 mM d-(+)-glucose). All the experiments were carried out in triplicate for a minimum of three times (*n* = 9). Data are expressed as mean ± SEM. * indicates significant statistical difference vs. vehicle (* *p* < 0.05; ** *p* < 0.01), while § indicates statistical difference vs. high glucose-treated cells pre-incubated with vehicle (§ *p* < 0.05; §§ *p* < 0.01; §§§ *p* < 0.001).

Treatment of vascular cells with HG induces an increase in intracellular ROS levels. In particular, in HASMCs, HG evoked an increase in ROS level by about 30% (% ROS vs. vehicle: 132.29 ± 5.23), while pre-incubation of erucin reduced ROS levels in a concentration-dependent manner (% ROS vs. vehicle in HASMCs treated with erucin 0.3 µM + HG: 108.29 ± 8.48, erucin 1 µM + HG: 95.53 ± 7.53; erucin 3 µM + HG: 93.99 ± 6.60) (Figure 4a). HG treatment in HUVECs evoked an increase in ROS by about 25% (% ROS vs. vehicle: 125.20 ± 4.71) and pre-incubation of erucin prevented, in a concentration-dependent manner, such an increase (% ROS vs. vehicle in HUVECs treated with erucin 0.3 µM + HG: 99.60 ± 8.28, erucin 1 µM + HG: 94.53 ± 4.64; erucin 3 µM + HG: 92.16 ± 4.06) (Figure 4b).

### 3.5. Erucin Inhibits HG-Induced Endothelial Hyperpermeability and Maintains Monolayer Integrity

**Figure 5:**
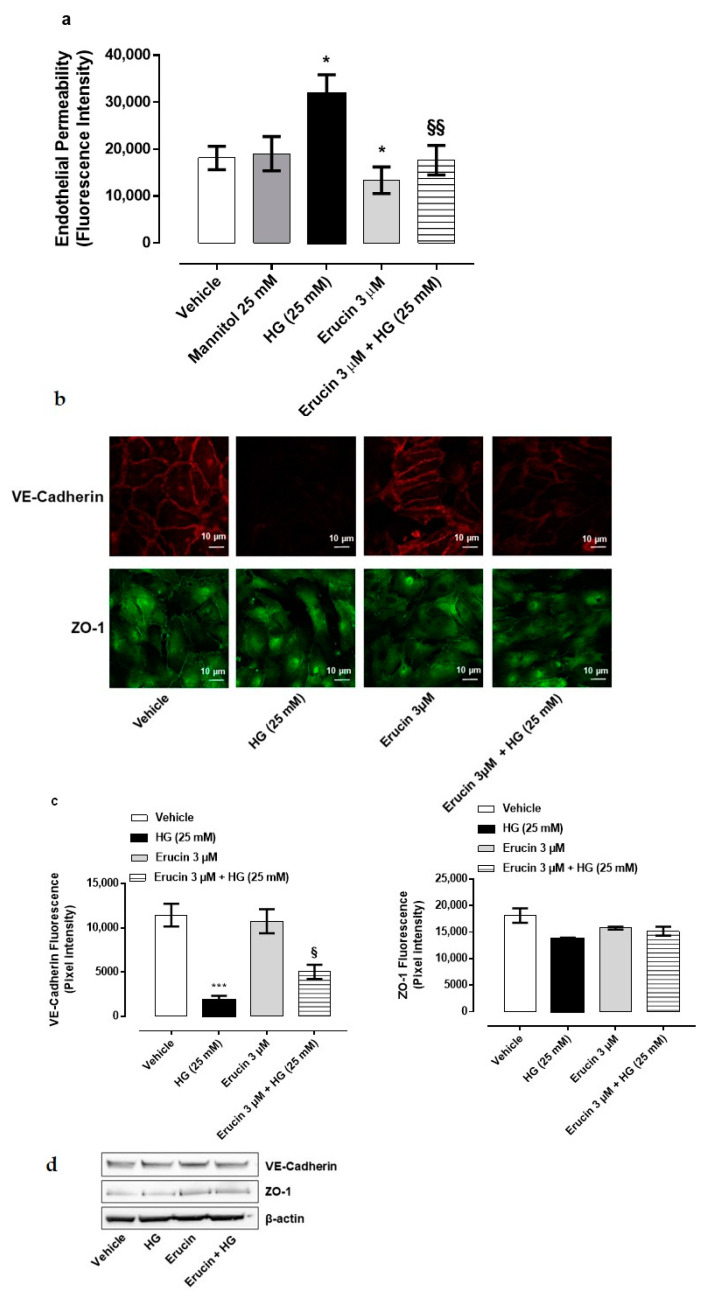
Effect of erucin on endothelial hyperpermeability induced by HG. Paracellular flux on HUVEC pre-treated with erucin (3 µM, 1 h) and then with HG (25 mM, 24 h) (**a**). FITC-dextran transport was measured at the end of HG stimulation. *n* = 3. * *p* < 0.05 vs. vehicle; §§ *p* < 0.01 vs. HG alone. (**b**). Integrity of cell–cell contacts by erucin evaluated by immunofluorescence analysis of VE-Cadherin (upper panels) and ZO-1 (lower panels) in HUVEC monolayers pre-treated with erucin (3 µM, 1 h) and then with high glucose (25 mM, 24 h). Images were obtained by confocal microscope. Scale bar—10 µm. (**c**). Fluorescence intensity was measured on four images per slide by using Fiji software. *** *p* < 0.001 vs. vehicle; § *p* < 0.05 vs. HG (25 mM). (**d**). Western blot analysis of VE-Cadherin and ZO-1 in HUVEC pre-treated with erucin (3 µM, 1 h) and then with HG (25 mM, 24 h).

High extracellular glucose concentrations are detrimental for endothelial cells and hyperglycemic conditions, such as diabetes mellitus, and increase endothelial cells permeability [27]. In order to investigate the contribution of erucin for endothelium integrity, a permeability assay was performed on confluent HUVEC monolayers. The HG condition significantly increased endothelial permeability, which was not due to tonicity of medium, since an equimolar concentration of mannitol showed a paracellular flux comparable with basal control (Figure 5a). The pre-incubation of endothelial monolayers with 3 µM erucin prevented HG-induced hyperpermeability and brought it back to the basal level, showing a protective effect *per se* (Figure 5a).

To further confirm our observations, immunofluorescence analysis of tight junction proteins in endothelial cells was also performed. VE-Cadherin and ZO-1 were evaluated as representative markers of endothelial tight junctions. HUVECs confluent monolayer expresses both proteins in control condition with a plasmalemmal localization at cell–cell contacts. Exposure to HG caused the fading of fluorescence intensity (Figure 5b,c), which was reverted by pre-treatment with erucin (3 µM). The effect was particularly evident with VE-cadherin, a typical endothelial marker. When cellular contents of tight junction proteins were analyzed, there was no change in cellular protein expression (Figure 5d). Collectively, these data indicate that erucin maintains the endothelial integrity and prevents the injury induced by HG on barrier function of endothelium.

### 3.6. Molecular Mechanisms Responsible for Endothelial Protection by Erucin

**Figure 6:**
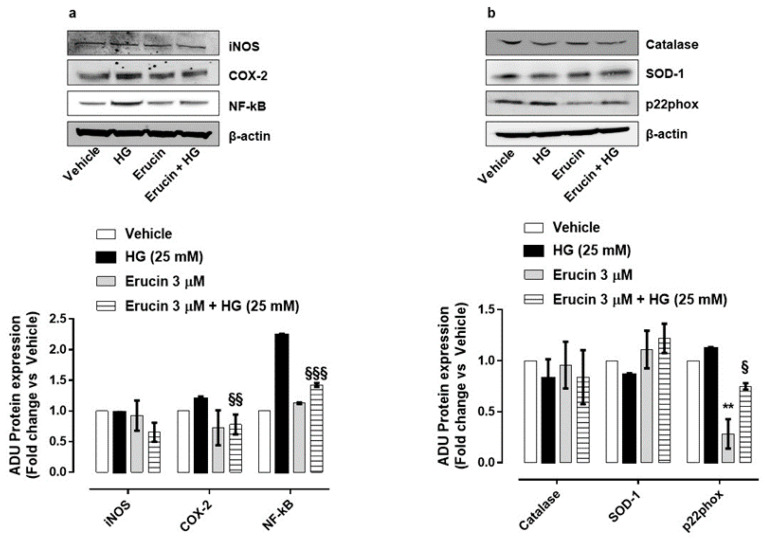
Effects of erucin on inflammatory and oxidative stress enzymes in endothelial cells treated with HG. Western blot analysis of inflammatory markers: iNOS, COX-2 and NF-κB (**a**) and oxidative/antioxidative enzymes: Catalase, SOD-1 and p22phox (**b**). Each lane contains 50 µg of total proteins obtained from endothelial cells pre-treated with erucin (3 µM, 1 h) and then with HG (25 mM, 24 h). The graphs represent the quantification of the optical density of each protein of interest respect to β-actin. ** *p* < 0.01 vs. vehicle; § *p* < 0.05 vs. HG (25 mM); §§ *p* < 0.01 vs. HG (25 mM); §§§ *p* < 0.001 vs. HG (25 mM).

The biochemical mechanisms associated with the protective effect of erucin on endothelium were then investigated. Key enzymes involved in inflammation and oxidative stress in response to HG were analyzed and the influence of erucin was investigated. The expression of the inducible cyclooxygenase-2 (COX-2), but not inducible nitric oxide synthase (iNOS), was prompted by HG and was drastically reduced by erucin pre-treatment (Figure 6a).

The ability of H_2_S to reduce nuclear factor kappa B (NF-κB) phosphorylation/activation under HG conditions has been already reported [28]. In our setting, HG enhanced NF-κB expression, which was significantly reduced by erucin pre-treatment (Figure 6a). Since HG induces a persistent oxidative status at the vascular level and isothiocyanates have been reported to exert antioxidant properties [29], cells were monitored for a panel of oxidant/antioxidant enzymes. HG caused a slight reduction in antioxidant superoxide dismutase-1 (SOD-1), which was reverted by erucin. Conversely, the expression of the subunit of NADPH oxidase p22phox was augmented in HUVEC exposed to HG and significantly reduced by erucin (Figure 6b). Interestingly, erucin significantly reduced, per se, p22phox expression. In contrast, catalase expression was not modified by HG or erucin (Figure 6b).

These data demonstrate the protective effect of erucin against inflammatory/pro-oxidative features at the endothelial level caused by hyperglycemia.

### 3.7. Anti-Inflammatory Effect of Erucin on Production of IL-6 and TNF-α

**Figure 7:**
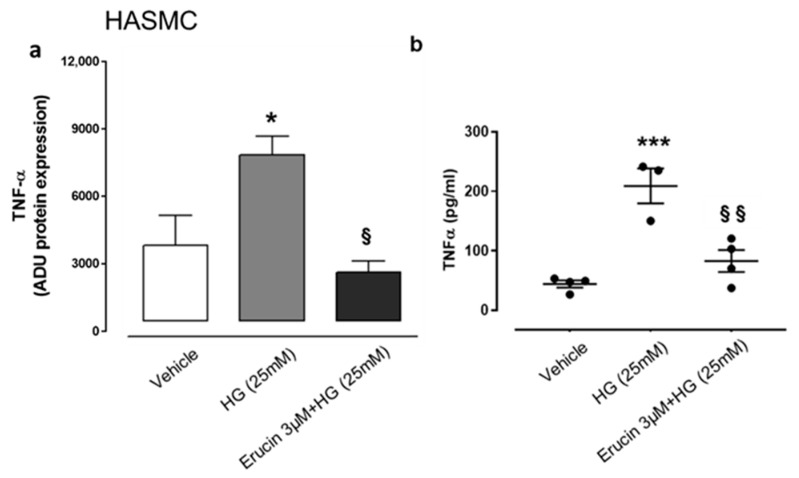
Effects of erucin on inflammatory cytokine in HASMCs treated with HG. TNF-α measurement through cytokine array in cell lysates (**a**) and ELISA quantification in cell supernatants (**b**). Each sample in (**a**) was used at a final concentration of 250 µg/mL. The graph in (**a**) represents the quantification of the optical density of TNF-α, standardized towards vehicle. * *p* < 0.05 vs. vehicle; *** *p* < 0.001 vs. vehicle; § *p* < 0.05 vs. HG (25 mM); §§ *p* < 0.01 vs. HG (25 mM).

**Figure 8:**
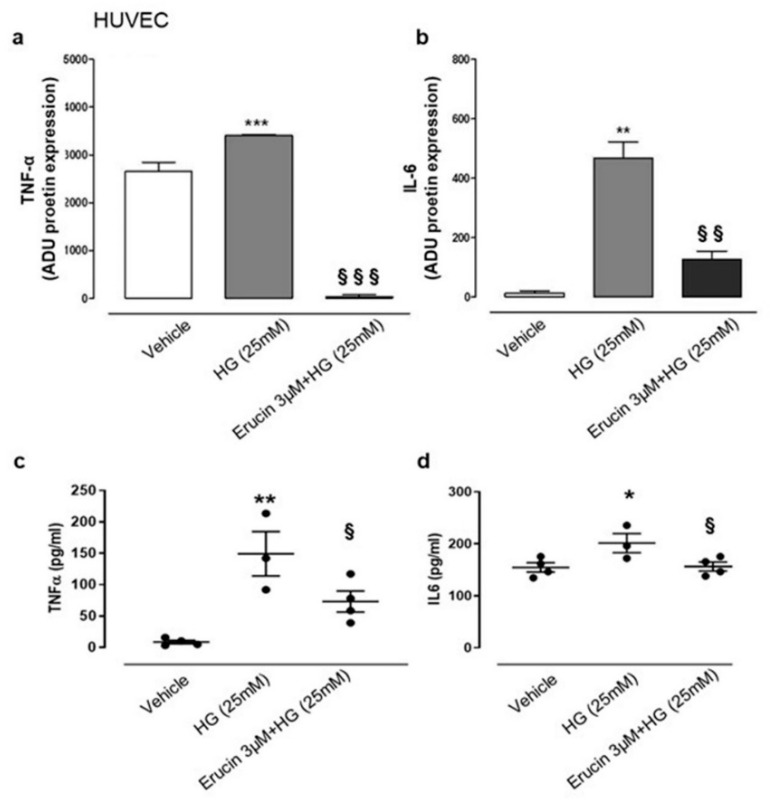
Effects of erucin on inflammatory cytokines in HUVECs treated with HG. TNF-α (**a**) and IL-6 (**b**) measurement in cell lysates through cytokine array. TNF-α (**c**) and IL-6 (**d**) ELISA quanti-fication in cell supernatants. Each sample in (**a**,**b**) was used at a final concentration of 250 µg/mL. The graphs in (**a**,**b**) represent the quantification of the optical density of cytokines of interest, standardized towards vehicle. * *p* < 0.05 vs. vehicle; ** *p* < 0.01 vs. vehicle; *** *p* < 0.001 vs. vehicle; § *p* < 0.05 vs. HG (25 mM); §§ *p* < 0.01 vs. HG (25 mM); §§§ *p* < 0.001 vs. HG (25 mM).

HG conditions affect physiology of vascular tissue and, given the possible beneficial effect of erucin in this experimental setting, we focused on possible modulation of inflammatory mediators in HASMCs and HUVECs. We evaluated the expression of different cytokines by using a specific array. We found that HG conditions in HASMCs specifically increased the expression of TNF-α (Figure 7), which was suppressed by the treatment with erucin (Figure 7a). These data were confirmed by ELISA determination, which quantified TNF-α in the same samples (Figure 7b). In addition, we also checked for differences in cytokine expression on HUVECs undergoing HG treatment. Similarly, we found a significant increase in expression of TNF-α together with IL-6 following HG exposure. Such an upregulation was suppressed by erucin treatment (Figure 8a,b). Again, these results were corroborated by quantification of cytokine levels through ELISA measurements, showing that erucin was effective in downregulating both TNF-α and IL-6, dampening the inflammation associated with HG exposure (Figure 8c,d).

## 4. Discussion

The pharmacological characterization of the protective activity of erucin on the vascular wall demonstrated that this isothiocyanate, derived from seeds of *Eruca sativa* Mill., is a H_2_S-donor able to release H_2_S inside the endothelial cells. The release of intracellular H_2_S induced by erucin was concentration-dependent, slow and gradual, i.e., similar to the endogenous H_2_S release. This suggests that erucin is a more suitable H_2_S donor compared to sulfide inorganic salt (i.e., NaHS, Na_2_S) to keep the vascular homeostasis and replace a possible deficiency in endogenous H_2_S production. The ability of erucin to release H_2_S could account for its protective/antioxidant effect at the vascular level. Indeed, H_2_S is recognized as both a direct and indirect antioxidant agent thanks to it being a reducing agent and its ability to activate fundamental antioxidant signaling pathways, such as the Nrf2/ARE pathway [10].

The vascular wall is the target of different detrimental stimuli. In particular, a dangerous condition identified as “vascular inflammation” results from chronic exposure of endothelium and vascular smooth muscle to high glucose levels, typical of diabetes. The exposure of both HUVECs and HASMCs to high levels of glucose induced a decrease in cell viability, an increase in endothelial cell permeability and an increase in inflammatory cytokines, thus possibly enhancing leukocyte recruitment at the endothelial level [30]. Erucin pre-incubation in the HG-induced cell injury exhibited a protective, concentration-dependent effect cell viability in HUVECs as well as HASMCs. This protective effect on endothelium and smooth muscle cells was also confirmed by the reduced activation of caspase 3/7, a typical marker of apoptosis, exerted by erucin.

HG-induced endothelial dysfunction is characterized by impaired vascular tone, redox imbalance and increased inflammatory reactions [31]. Erucin reduced the inflammatory environment in endothelial cells by downregulation of COX-2 expression prompted by HG, while there was no interference with iNOS expression. In endothelial cells, H_2_S attenuated the inflammation response through the inhibition of the transcriptional factor NF-κB [32]. Consistently, pre-treatment of HUVECs with erucin reduced the activation of NF-κB mediated by HG, establishing the potential use of erucin to counteract inflammatory stimuli.

The phosphorylation of cell–cell contact proteins mediated by PKC plays a critical role in increasing the permeability of diabetic endothelium. Indeed, hyperglycemia is a primary factor involved in endothelial barrier dysfunction, through the phosphorylation of VE-Cadherin and disruption of endothelial junctions [33]. Erucin was able to reduce HG-induced hyperpermeability and resumed disruption of cell–cell contact markers (VE-Cadherin and ZO-1) after HG treatment. Such an erucin-induced activity is possibly due to the maintenance of adherence, expression and localization of tight junction proteins, not excluding an involvement of the transcription factor NF-κB.

The exposure to high glucose levels induced not only an increase in inflammatory markers but also an increase in a typical marker of oxidative stress, i.e., ROS. Erucin significantly prevented the increase in ROS levels both in HUVECs and in HASMCs in a concentration-dependent manner. The antioxidant effect of erucin is likely to be related not only to the direct scavenging activity of intracellularly released H_2_S but also to the change in the expression and activity of pro-/antioxidant enzymes. Indeed, in endothelial cells, erucin reduced the expression of pro-oxidant marker p22phox and mildly increased the expression of the antioxidant enzyme SOD-1. These results are consistent with ROS burden reduction in HUVEC treated with erucin and exposed to HG.

Although ROS generation is one of the key events following hyperglycemia, the knowledge about the role of blood cells–endothelium–smooth muscle interplay in driving the inflammatory process is now a cornerstone of inflammatory based vascular disease [1,34]. In this respect, the effect of erucin in controlling expression of key inflammatory mediators such as TNF-α and IL-6 is consistent with the anti-inflammatory role of H_2_S [35,36]. Indeed, H_2_S released by erucin could reduce leukocyte trafficking through the vascular wall and this effect can occur through reduction in adhesion molecules expression associated with low levels of TNF-α and IL-6 [37,38].

The use of freshly isolated and cultured cells allowed us to investigate many signaling pathways relevant to human physiology and pathophysiology. However, as a limitation of this study, the phenotype of vascular endothelial and smooth muscle cells with respect to ion channels, receptors, calcium store mechanisms, accessory proteins and other vascular functions may vary within and between vascular beds in health and disease. In addition, further variables that can be acknowledged are development, ageing and cell isolation/culture procedures (including different lab techniques).

Nevertheless, HUVEC and HASMC cell lines represent widely used and reliable experimental tools for pharmacological testing of novel compounds with potential vascular effects. Indeed, endothelial cells, as well as smooth muscle cells, represent the first lines to be involved in the inflammatory process that could drive possible detrimental effects in underlying tissue, if not well-controlled. This work demonstrated that erucin, in HUVEC and HASMC cell lines, can control inflammation in hyperglycemic conditions through different mechanisms, all aiming to reduce the damage in human endothelial as well as smooth muscle cultured cells. These findings lead us to hypothesize a potential role for erucin in preventing vascular disease associated with hyperglycemia.

## 5. Conclusions

Vascular inflammation represents a pathological condition which has received appropriate consideration in the last few years. Indeed, only recently this condition has emerged as the first step of a degenerative process which firstly attacks the vascular wall and then spreads inflammation in several tissues. The possibility to stem the diffusion of subclinic chronic inflammation to organs and systems is strictly connected with the ability to preserve the integrity of the vascular wall, and, in particular, of the first layer which is exposed to the assault of ox-inflamm-aging process, i.e., the endothelium. Indeed, ageing and the co-existing metabolic diseases, such as metabolic syndrome, diabetes, dyslipidemia and obesity, induce the persistence of oxidative and inflammatory stimuli on the vessels and in particular on the endothelial cells. This leads to the disruption of the “barrier function” played by the endothelial layer and dramatically alters the role of “chemical laboratory” of endothelial cells. In this panorama, the preservation of the vascular wall function and integrity represents a compelling challenge for therapeutical and nutraceutical approaches. This preliminary study demonstrates the ability of erucin in counteracting the increase in oxidative and inflammatory markers in human endothelial and smooth muscle cells exposed to a high glucose stimulus. Moreover, this work also confirms the ability of erucin to potentially preserve the integrity of the vascular wall by limiting endothelial permeability. Therefore, the isothiocyanate erucin could be seen as a therapeutical/nutraceutical opportunity to limit the onset of the vascular inflammation condition.

## Data Availability

The data presented in this study are available in this article.
